# Minimum triplet covers of binary phylogenetic *X*-trees

**DOI:** 10.1007/s00285-017-1117-6

**Published:** 2017-06-12

**Authors:** K. T. Huber, V. Moulton, M. Steel

**Affiliations:** 1 School of Computing Sciences, University of East Anglia, Norwich, UK; 2 Biomathematics Research Centre, University of Canterbury, Christchurch, New Zealand

**Keywords:** Trees, Median vertex, 2-Trees, Shellability, Reconstruction, 94C15, 68R10, 05C05, 05C99, 05C62

## Abstract

Trees with labelled leaves and with all other vertices of degree three play an important role in systematic biology and other areas of classification. A classical combinatorial result ensures that such trees can be uniquely reconstructed from the distances between the leaves (when the edges are given any strictly positive lengths). Moreover, a linear number of these pairwise distance values suffices to determine both the tree and its edge lengths. A natural set of pairs of leaves is provided by any ‘triplet cover’ of the tree (based on the fact that each non-leaf vertex is the median vertex of three leaves). In this paper we describe a number of new results concerning triplet covers of minimum size. In particular, we characterize such covers in terms of an associated graph being a 2-tree. Also, we show that minimum triplet covers are ‘shellable’ and thereby provide a set of pairs for which the inter-leaf distance values will uniquely determine the underlying tree and its associated branch lengths.

## Introduction

Trees play a central role in systematic biology, and other areas of classification, such as linguistics. It is often assumed that such a tree *T* has a labelled leaf set *X*, that all vertices have degree 1 or at least three, and that there is an assignment of a positive real-valued length to each edge of *T*.

A classical and important result from the 1960s and 1970s asserts that any such tree *T* with edge lengths is uniquely determined from the induced leaf-to-leaf distances between each pair of elements of *X*. This result is the basis of widely-used methods for inferring trees from distance data, such as the popular ‘Neighbor-Joining’ algorithm (Saitou and Nei 1987). Moreover, when *T* is binary (each non-leaf vertex has degree 3) then we do not require distance values for all of the $$\left( {\begin{array}{c}n\\ 2\end{array}}\right) $$ pairs from *X* (where $n=|X|$), since just $2n-3$ carefully selected pairs of leaves suffice to determine *T* and its edge lengths [see Guénoche et al. (2004); more recent results appear in Dress et al. (2012), motivated by the irregular distribution of genes across species in biological data].

This value of $2n- 3$ cannot be made any smaller, since a binary unrooted tree with *n* leaves has $2n-3$ edges, and the inter-leaf distances are linear combinations of the corresponding $2n-3$ edge lengths (so, by linear algebra, these values cannot be uniquely determined by fewer than $2n-3$ equations).

There is a particularly natural way to select a subset of $$\left( {\begin{array}{c}X\\ 2\end{array}}\right) $$ for *T* when *T* is binary. Since each non-leaf vertex is incident with three subtrees of *T*, let us (i) select a leaf from each subtree, (ii) consider the three pairs of leaves we can form from this triple, and then (iii) take the union of these sets of pairs over all non-leaf vertices of *T*. This process produces a ‘triplet cover’ of *T* (defined more precisely below).

A triplet cover need not be of this minimum size (i.e. of size $2n-3$) but in this paper we characterize when it is. Also, we show that in that case the resulting triplet cover is ‘shellable’ which implies that the inter-leaf distances defined on these pairs uniquely determine the tree and its edge lengths. These, and other results obtained along the way complement recent work into phylogenetic ‘lasso’ sets (Dress et al. 2012; Huber and Steel 2014), as well as a Hall-type characterization of the median function on trees in Dress and Steel (2009).

We begin with some definitions.

### Definitions

Let *X* be a finite set with $|X| \ge 3$. We denote elements in $X \atopwithdelims ()2$ and $X \atopwithdelims ()3$ also by *ab* and *abc*, respectively, where $a,b,c \in X$ are distinct. We refer to the elements in ${X\atopwithdelims ()3}$ as *triples*.

A *(binary) phylogenetic* *X*-*tree* is an unrooted tree $T=(V,E)$ which has leaf set *X*, and for which each non-leaf vertex is unlabelled and of degree three. We let *B*(*X*) denote the set of binary phylogenetic *X*-trees (two such trees are regarded as equivalent if there is a graph isomorphism between them that maps leaf *x* in one tree to leaf *x* in the other tree, for all $x \in X$). In evolutionary biology, the set *X* usually corresponds to some collection of species or taxa.

Note that a phylogenetic *X*-tree *T* must contain at least one *cherry* $\{a,b\}$, that is, *a* and *b* are adjacent with the same interior vertex of *T*. Moreover, if $|X|>3$ then each tree $T \in B(X)$ has at least two cherries that are vertex disjoint from each other; if *T* has exactly two cherries we say it is a *caterpillar* tree [every tree in *B*(*X*) is a caterpillar when $|X|=4$ or $|X|=5$]. When $|X|=4$, we say that $T \in B(X)$ is a *quartet*, and if the two cherries of this tree are (say) $\{a,b\}$ and $\{c,d\}$ then we denote *T* by *ab*|*cd*.

We let ${V}={V}(T)\subseteq V$ denote the set of $|X|-2$ interior vertices of *T*. Given $x \in X$ where $|X|\ge 4$, we let $T-x$ denote the phylogenetic $(X-\{x\})$-tree which is obtained by removing the leaf *x* (and its incident edge) from *T* and suppressing the resulting degree 2 vertex.

**Fig. 1:**
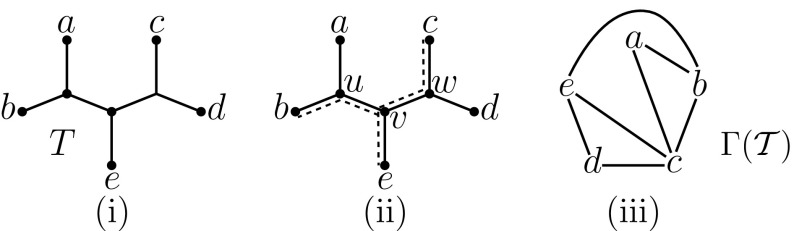
**i** A tree $T \in B(X)$ for $X=\{a,b,c,d,e\}$; **ii** vertex *v* is supported by the triple *bce* (the *dashed lines* show the edge-disjoint paths from *v* to these three leaves); **iii** the cover graph $\Gamma (\mathcal {T})$ corresponding to the triplet cover $\mathcal {T}$ obtained by taking all pairs from the triple *bce* that supports *v* and from the triples *abc* and *cde* that support vertices *u* and *w*, respectively. This triplet cover is minimal, and since its size is 7 ($=2n-3$ for $n=|X|$) it is also a minimum triplet cover for the tree (by Proposition 3)

Suppose that $\mathcal {T}$ is a subset of $X \atopwithdelims ()2$, and $T=(V,E) \in B(X)$. We say that a triple in ${X \atopwithdelims ()3}$ *supports* a vertex $v \in {V}$ in *T* (relative to $\mathcal {T}$) if we can select leaves $a,b,c \in X$, one from each connected component of the graph obtained by removing *v* and its incident edges from *T*, such that $ab, ac, bc \in \mathcal {T}$. We call a subset $\mathcal {T}\subseteq {X \atopwithdelims ()2}$ a *triplet cover* for *T* if for each vertex $v\in {V}$ there is some triple in ${X \atopwithdelims ()3}$ that supports *v* (relative to $\mathcal {T}$). Note that $X=\bigcup _{A \in \mathcal {T}} A$ holds in this case. Given a non-empty subset $\mathcal {T}\subseteq {X \atopwithdelims ()2}$, we define the *cover graph* $\Gamma (\mathcal {T})=(X,\mathcal {T})$ (*of* $\mathcal {T}$) to be the graph with vertex set *X* and edge set $\mathcal {T}$.

We illustrate these concepts in Fig. 1. For the binary phylogenetic *X*-tree in Fig. 1i (with $X=\{a, \ldots , e\}$) the vertex *v* (in Fig. 1ii) is supported by the triple *bce* (there are three other triples that support *v*). If *u* is supported by, say, *abc* and *w* by *cde* then we obtain the triplet cover

$$\begin{aligned} \mathcal {T}= \left( {\begin{array}{c}\{b,c,e\}\\ 2\end{array}}\right) \cup \left( {\begin{array}{c}\{a,b,c\}\\ 2\end{array}}\right) \cup \left( {\begin{array}{c}\{c,d,e\}\\ 2\end{array}}\right) = \{ab, ac, bc, cd, ce, de, be\}. \end{aligned}$$

The corresponding cover graph $\Gamma (\mathcal {T})$ is shown in Fig 1iii.

Given a tree $T\in B(X)$, a triplet cover $\mathcal {T}$ for *T* is called

- *minimal* if $\mathcal {T}-\{ab\}$ is not a triplet cover for *T*, for any $ab \in \mathcal {T}$;
- *minimum* if $|\mathcal {T}| \le |\mathcal {T}'|$ for every triplet cover $\mathcal {T}'$ for *T*.

These two concepts are different; there exist minimal triplet covers that are not minimum (we describe an example in the final section).

Note that it can be shown that any minimum triplet cover on *X* must have cardinality $2|X|-3$ [by applying Theorem 1 and Proposition 1 of Dress et al. (2012)]. Moreover, there are various ways to construct triplet covers that are minimum [for example, ‘pointed covers’ (Dress et al. 2012, Theorem 7) and ‘stable triplet covers’ (Huber and Steel 2014, Theorem 1)].

### Outline of main results

In this paper, we prove a structural result concerning minimum triplet covers. Namely, we prove that a set $$\mathcal {T}\subseteq \left( {\begin{array}{c}X\\ 2\end{array}}\right) $$ is a minimum triplet cover for a tree $T \in B(X)$ if and only if the associated cover graph $\Gamma (\mathcal {T}) = (X,\mathcal {T})$ is a 2-tree (see Theorem 1 and Sect. 5 for the definition of a 2-tree).

Using the concepts that we develop to prove this result, we also give an independent proof [that does not require the notion of phylogenetic ‘lassos’ from Dress et al. (2012)] that any minimum triplet cover on *X* must have cardinality $2|X|-3$ (Proposition 3). As a corollary of our structural result, we also show that if $\mathcal {T}$ is a minimum triplet cover for *T* then it is shellable for *T* (Proposition 4).

This corollary has two important implications. First it implies [from results in Dress et al. (2012)] that if $\mathcal {T}$ is a minimum triplet cover for *T*, then *T* (together with its edge lengths) can be uniquely reconstructed from the tree metric restricted to the pairs in $\mathcal {T}$. Note that this can also be deduced from results in Leclerc and Makarenkov (1998) that relate 2-trees and tree metrics [see also Guénoche et al. (2004)].

Second, the corollary gives an independent proof of Dress et al. (2012), Theorem 7 and Huber and Steel (2014), Theorem 1 which state that pointed triplet covers and stable triplet covers are shellable, respectively.

## The support graph

In this section we introduce a graph that can be associated to a triplet cover of a tree. Properties of this graph will be used to help prove our results later on. We begin with some further definitions.

Suppose for the following that $T=(V,E) \in B(X)$. Given a subset $\mathcal {T}\subseteq {X \atopwithdelims ()2}$ and $v \in {V}$, we let $S_v(\mathcal {T})$ be the subset of $X \atopwithdelims ()3$ which contains precisely those triples in ${X\atopwithdelims ()3}$ that support *v* (relative to $\mathcal {T}$). We call $S_v(\mathcal {T})$ the *support of* *v* (*relative to* $\mathcal {T}$). In addition, suppose that $a,b,c\in V$ are pairwise distinct. Then we call the unique vertex of *T* that simultaneously lies on the shortest path from *a* to *b*, from *b* to *c*, and from *a* to *c* the *median* of *a*, *b*, and *c*, denoted by $\mathrm{med}_T(a,b,c)$. The following observation linking medians with supports will be useful.

### Lemma 1

Let $T=(V,E)\in B(X)$ and $\mathcal {T}\subseteq {X \atopwithdelims ()2}$. If $abc \in S_v(\mathcal {T})$, $v \in {V}$, then $v = \mathrm{med}_T(a,b,c)$. Moreover, $\mathcal {T}$ is a triplet cover of *T* if and only if $|S_v(\mathcal {T})| \ge 1$ for all $v \in {V}$.

Now, given a non-empty subset $\mathcal {T}\subseteq {X \atopwithdelims ()2}$ and some $x \in X$, we put

$$\begin{aligned} \mathcal {T}^{-x} = \mathcal {T}- \{ xa \,: \, a \in X-\{x\} \text{ and } xa \in \mathcal {T}\}. \end{aligned}$$

**Fig. 2:**
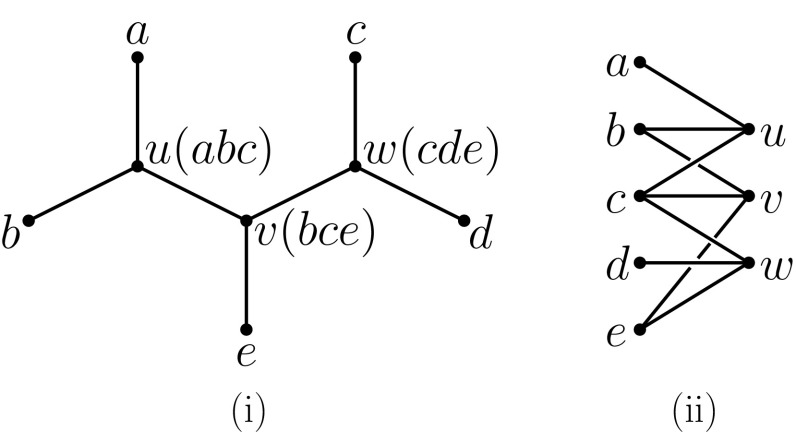
For triplet cover $\mathcal {T}$ for the example from Fig. 1 reproduced in (**i**), with the triple supporting an interior vertex shown in parentheses, the corresponding support graph $G(\mathcal {T})$ is shown in (**ii**)

Put differently, $\mathcal {T}^{-x}$ is the subset of $\mathcal {T}$ obtained by removing from $\mathcal {T}$ precisely those elements in $\mathcal {T}$ which contain *x*. We also define a bipartite graph $G(\mathcal {T}) = (X \amalg {V}, E(\mathcal {T}))$, with edge $\{x,v\} \in E(\mathcal {T})$, $x \in X$, $v \in {V}$, if $x \in A$ for all $A \in S_v(\mathcal {T})$. We call $G(\mathcal {T})$ the *support graph* associated to $\mathcal {T}$. For any vertex *p* of $G(\mathcal {T})$, we let $\deg _{\mathcal {T}}(p)=\deg _{G(\mathcal {T})}(p)$ denote the degree of *p* in $G(\mathcal {T})$. In Fig. 2ii we illustrate the support graph for the triplet cover $\mathcal {T}$ given in Fig. 1.

We now list some properties of $G(\mathcal {T})$.

### Proposition 1

Suppose that $\mathcal {T}$ and $\mathcal {T}'$ are triplet covers of a tree $T=(V,E) \in B(X)$, and that $x \in X$.

1. If $v \in {V}$, then $0 \le \deg _{\mathcal {T}}(v) \le 3$, and $1 \le \deg _{\mathcal {T}}(x) \le |X|-2$.
2. If $\mathcal {T}' \subseteq \mathcal {T}$, then $E(\mathcal {T}) \subseteq E(\mathcal {T}')$. In particular, if there exists some $x \in X$ with $\deg _{\mathcal {T}'}(x)=1$, then $\deg _{\mathcal {T}}(x)=1$.
3. If $\mathcal {T}$ is a minimal triplet cover for *T*, then for all $ab \in \mathcal {T}$, there exists some $v \in {V}$ such that *a*, *v*, *b* is a path in $G(\mathcal {T})$.
4. Suppose that *v* is the vertex adjacent to *x* in *T*. Then $\{v,x\} \in E(\mathcal {T})$. Furthermore $\deg _{\mathcal {T}}(x) = 1$ if and only if $\{v,x\}$ is the only edge in $G(\mathcal {T})$ that contains *x*.
5. $\deg _{\mathcal {T}}(x) = 1$ if and only if $\mathcal {T}^{-x}$ is a triplet cover of $T-x$.
6. If $$\deg _{\mathcal {T}}(x) = 1$$, then $$|\mathcal {T}| \ge |\mathcal {T}^{-x}|+2$$.

### Proof

1. The inequality $\deg _{\mathcal {T}}(v) \le 3$ follows immediately from the definition of the support $S_w(\mathcal {T})$ of a vertex $w\in {V}$ and the fact that *T* is binary. The inequality $1 \le \deg _{\mathcal {T}}(x)$ follows since $x \in A$ for all $A \in S_u(\mathcal {T})$ for the vertex *u* that is adjacent to *x* in *T*. The inequality $\deg _{\mathcal {T}}(x) \le |X|-2$ follows from the fact that $T \in B(X)$ and so has $|X|-2$ interior vertices.
2. Suppose that $\{v,x\} \in E(\mathcal {T})$, $x \in X, v \in {V}$. Then $x \in A$, for all $A \in S_v(\mathcal {T})$. Since $S_v(\mathcal {T}')\subseteq S_v(\mathcal {T})$ as $\mathcal {T}'\subseteq \mathcal {T}$ it follows that $x \in A$ for all $A \in S_v(\mathcal {T}')$. Hence, $\{v,x\} \in E(\mathcal {T}')$. The second statement is a trivial consequence in light of the inequality $1 \le \deg _{\mathcal {T}}(x)$ from (P1).
3. Suppose for contradiction that there exists some $ab\in \mathcal {T}$ such that for all $v\in {V}$, we have that *a*, *v*, *b* is not a path in $G(\mathcal {T})$. Then for all $v\in {V}$ there must exist some $A\in S_v(\mathcal {T})$ such that $ab\not \subseteq A$. Hence, $\mathcal {T}'=\mathcal {T}-\{ab\}$ is a triplet cover of *T*. Since $\mathcal {T}'\subsetneq \mathcal {T}$ clearly holds, we obtain a contradiction in view of the minimality of $\mathcal {T}$.
4. That $\{v,x\}\in E(\mathcal {T})$ holds is an immediate consequence of the choice of *v*. If $\deg _{\mathcal {T}}(x) =1$, then since $x \in A$ for all $A \in S_v(\mathcal {T})$, it follows that $\{x,v\}$ is in $E(\mathcal {T})$. The rest of the statement follows immediately.
5. Suppose that $$\mathcal {T}^{-x}$$ is not a triplet cover of $$T-x$$. Then, by Lemma 1, there exists an interior vertex *u* of $$T-x$$ such that $$S_u(\mathcal {T}^{-x})=\emptyset $$. Let $$u' $$ be the vertex in *T* that corresponds to *u* in $$T-x$$. Then as $$S_u(\mathcal {T}^{-x})=\emptyset $$, it follows that $$x \in A$$ for all $$A \in S_{u'}(\mathcal {T})$$. Hence $$\{x,u'\}\in E(\mathcal {T})$$ and, so, $$\deg _{\mathcal {T}}(x)\ge 1$$. Moreover, if *v* is the vertex adjacent to *x* in *T*, then $$v \ne u'$$. By (P4), it follows that $$\{x,v\}$$ is also an edge in $$E(\mathcal {T})$$. Therefore $$\deg _{\mathcal {T}}(x)>1$$.
  Conversely, suppose that $\mathcal {T}^{-x}$ is a triplet cover for $T-x$, and assume for contradiction that $\deg _{\mathcal {T}}(x) \ge 2$. Then there exist $u,v \in {V}$ distinct such that $x \in A$ for all $A \in S_u(\mathcal {T})$ and $x \in B$ for all $B \in S_v(\mathcal {T})$. Without loss of generality, we may assume that *v* is the vertex in *T* that is adjacent to *x*. Let $u'$ be the vertex in $T-x$ that corresponds to *u* in *T*. Then $S_{u'}(\mathcal {T}^{-x})=\emptyset$ since $x\in A$ for all $A \in S_u(\mathcal {T})$. Hence $\mathcal {T}^{-x}$ is not a triplet cover for $T-x$, a contradiction.
6. If *v* is the vertex in *T* adjacent to *x*, then $S_v(\mathcal {T}) \ne \emptyset$ by Lemma 1. Hence, there must be some $A \in S_v(\mathcal {T})$ with $x \in A$. But then $|\mathcal {T}- \mathcal {T}^{-x}|\ge 2$.$\square$

We now show that any minimal triplet cover of a tree in *B*(*X*) has a size that grows linear with |*X*|.

### Corollary 1

Suppose that $\mathcal {T}$ is a minimal triplet cover of some $T\in B(X)$. Then

$$\begin{aligned} |\mathcal {T}| \le 3(|X|-2). \end{aligned}$$

### Proof

Put $T=(V,E)$. First we observe that if $B=(X \amalg {V},E')$ is a bipartite graph in which every vertex in ${V}$ has degree at most 3, then the number of length 2 paths in *B* of the form *x*, *v*, *y* with $x,y \in X$ and $v \in {V}$ is equal to

$$\begin{aligned} \sum _{v \in {V}} | \{x, v, y \,:\, x,y \in X \text{ and } x, v, y \text{ a } \text{ path } \text{ in } B \}|. \end{aligned}$$

Now, by (P3), $|\mathcal {T}|$ is less than or equal to the number of length 2 paths in $G(\mathcal {T})$ of the form *x*, *v*, *y* with $x,y \in X$ and $v \in {V}$. Since $|{V}|=|X|-2$, and each term in the above sum is at most 3 the corollary follows. $\square$

## Multiplicities

In this section we derive some bounds for degrees of vertices in the cover graph of a triplet cover. Suppose that $\mathcal {T}$ is a triplet cover of $T \in B(X)$. For $x \in X$ we define the *multiplicity* $\mu (x)=\mu _{\mathcal {T}}(x)$ *of* *x* (*relative to* $\mathcal {T}$) to be the number of elements in $\mathcal {T}$ that contain *x* [or in other words, the degree of the vertex *x* in the cover graph $\Gamma (\mathcal {T})$]. The *multiplicity of* $\mathcal {T}$ is $\mu (\mathcal {T}) = \min \{\mu _{\mathcal {T}}(x): x\in X\}$.

The following observation relating multiplicities with degrees will be useful later.

### Lemma 2

Suppose that $\mathcal {T}$ is a triplet cover for some tree $T\in B(X)$ and $x \in X$. If $\mu (x)=2$, then $\deg _{\mathcal {T}}(x)=1$.

### Proof

If $\mu (x)=2$, then *x* can be contained in at most one element of $\bigcup _{v \in {V}} S_v(\mathcal {T})$. But *x* must be contained in every element of $S_u(\mathcal {T})$ for *u* the vertex in ${V}$ that is adjacent to *x* in *T*. Hence $|S_u(\mathcal {T})|=1$, and the only edge contained in the support graph $G(\mathcal {T})$ that contains *x* (which must exist by (P1)) is $\{x,u\}$. In particular, $\deg _{\mathcal {T}}(x)=1$. $\square$

We now derive some bounds for multiplicities of minimal and minimum triplet covers.

### Proposition 2

Suppose that $$T\in B(X)$$.

1. If $\mathcal {T}$ is a minimal triplet cover for *T*, then $2 \le \mu (\mathcal {T}) \le 5$.
2. If $\mathcal {T}$ is a minimum triplet cover for *T*, then $2 \le \mu (\mathcal {T}) \le 3.$

### Proof

*(M1):* Suppose that $x \in X$. Let *v* be the vertex in *T* adjacent to *x* in *T*. Then, as $\mathcal {T}$ is a triplet cover for *T*, by Lemma 1 there must exist some $axy \in S_v(\mathcal {T})$ where $a,y \in X-\{x\}$ are distinct. Therefore $2 \le \mu (x)$ for all $x \in X$ and so $2 \le \mu (\mathcal {T})$.
  To see that the remaining inequality holds, we show that there is some element of *X* that is contained in at most 5 elements of $\mathcal {T}$. We use a simple counting argument based on pairs (*x*, *c*) where $x \in X$ is an element in some $c \in \mathcal {T}$. By Corollary 1, $|\mathcal {T}|\le 3(|X|-2)$ as $\mathcal {T}$ is minimal. Since each element of $\mathcal {T}$ contains 2 elements of *X*, the size of the set *R* of pairs (*x*, *c*) is at most $6(|X|-2)$. On the other hand $\sum _{x \in X} \mu (x) = |R|$. Hence, since $|X|\ge 3$, there must exist some $x \in X$ with $\mu (x) \le 5$.
*(M2):* We again count pairs (*x*, *c*) where $x \in c$ and *c* is an element in $\mathcal {T}$. This is $2 |\mathcal {T}| = 2(2|X|-3)$ and also equal to $\sum _{x \in X} \mu (x)$. Since $2(2|X|-3)<4|X|$ and $|X|\ge 3$, there is some $x \in X$ with $\mu (x) \le 3$. That $\mu (\mathcal {T})\ge 2$ holds follows from (M1).$\square$

## A lower bound

In this section, we show that a minimum triplet cover of a tree $T \in B(X)$ has size $2|X|-3$. As mentioned in the introduction, this result can also be derived by applying Theorem 1 and Proposition 1 of Dress et al. (2012). However, it is of interest to have a direct proof that is independent of results concerning tree metrics.

### Proposition 3

Suppose that $\mathcal {T}$ is a triplet cover for some $T \in B(X)$. Then we have $|\mathcal {T}| \ge 2|X|-3$. Moreover this bound is tight.

### Proof

We use induction on $n =|X|$. The result clearly holds for $n=3$. So, suppose that the result holds for all triplet covers of trees in *B*(*X*) with $3 \le |X| \le n-1$.

Suppose that $\mathcal {T}$ is a triplet cover for a tree in *B*(*X*) with $|X|=n$. If there exists some $a \in X$ such that $\deg _{\mathcal {T}}(a)=1$, then by (P5) $\mathcal {T}^{-a}$ is a triplet cover for $T-a$. Hence, by (P6) and induction, $|\mathcal {T}| \ge |\mathcal {T}^{-a}| + 2 \ge 2n-3$.

So, suppose that $\deg _{\mathcal {T}}(x)\ge 2$ for all $x \in X$. Note that there must exist some $a \in X$ with $\deg _{\mathcal {T}}(a)=2$ (otherwise, $\deg _{\mathcal {T}}(x)\ge 3$ for all $x \in X$ implies that there is a vertex $v \in {V}$ with $\deg _{\mathcal {T}}(v)\ge 4$, which contradicts (P1)). Suppose that $v,u \in {V}$ are distinct with $\{a,v\}, \{a,u\}$ in $E(\mathcal {T})$. Then there exist distinct elements $b,c,x,y \in X-\{a\}$ with $\{b,x\} \ne \{c,y\}$ such that $abx \in S_v(\mathcal {T})$ and $acy \in S_u(\mathcal {T})$. Put $C:=\{b,x\}\cap \{c,y\}$. Then since $\{b,x\} \ne \{c,y\}$ it follows that $|C|<2$ and so we consider the two possible cases ($|C|=1$ and $|C|=0$).

*Case 1:* $|C|=1$. Without loss of generality we may assume $x=c$ and $y \ne b$. Then it is straight-forward to see that without loss of generality, *v* is adjacent to *a* in *T*, *u* lies on the path in *T* between *v* and *c*, and *T* restricted to the set $\{a,b,c,y\}$ is the quartet *ab*|*cy*. Note that $by \not \in \mathcal {T}$ since otherwise $bcy \in S_u(\mathcal {T})$ which contradicts $\{a,u\} \in E(\mathcal {T})$.
  Consider the triplet cover $\mathcal {T}' = \mathcal {T}\cup \{by\}$ of *T*. Then $acy,bcy \in S_u(\mathcal {T}')$. Hence, since $E(\mathcal {T}')\subseteq E(\mathcal {T})$ by (P2), $\deg _{\mathcal {T}'}(a)=1$. Therefore, by (P5), $\mathcal {T}'^{-a}$ is a triplet cover of $T-a$. But the elements *ab*, *ac*, *ay* of $\mathcal {T}$ are not contained in $\mathcal {T}'^{-a}$ and, so,
  $$\begin{aligned} |\mathcal {T}'^{-a}|+3 \le |\mathcal {T}'| = |\mathcal {T}|+1. \end{aligned}$$
  The fact that $|\mathcal {T}| \ge 2|X|-3$ holds now follows immediately by induction.
*Case 2:* $|C|=0$. Then $x \ne c$ and $y \ne b$. Without loss of generality, we can assume that *v* is adjacent to *a* in *T*, and that *T* restricted to the set $\{a,b,c,y,x\}$ is a caterpillar tree with cherry $\{a,x\}$. We consider the case where $\{y,c\}$ is also a cherry in this caterpillar tree and *u* is adjacent to both *y* and *c* in *T*. The argument for the remaining case (where $\{b,y\}$ or $\{b,c\}$ is also a cherry) is similar.

First note that if $bc \in \mathcal {T}$, then $by \not \in \mathcal {T}$, since otherwise $byc \in S_u(\mathcal {T})$ which would contradict $\{a,u\} \in E(\mathcal {T})$. Similarly if $cx \in \mathcal {T}$, then $yx \not \in \mathcal {T}$. Hence, by symmetry, we can assume that $\mathcal {T}$ does not contain at least one element from the set $\{bc, by\}$ and at least one element from the set $\{cx,yx\}$. Now, let *P* be a subset of $\{bc,by,cx,yx\}-\mathcal {T}$ of minimum size such that $\mathcal {T}\cup P$ contains precisely one of the sets $\{bc, by\}$ or $\{cx,yx\}$, noting that $|P|\le 2$. Consider the triplet cover $\mathcal {T}' = \mathcal {T}\cup P$ of *T*. Then it is easily seen that $\deg _{\mathcal {T}'}(a)=1$, and so by (P5) $\mathcal {T}'^{-a}$ is a triplet cover of $T-a$. But the elements *ab*, *ac*, *ax*, *ay* of $\mathcal {T}$ are not contained in $\mathcal {T}'^{-a}$ and so

$$\begin{aligned} |\mathcal {T}'^{-a}|+4 \le |\mathcal {T}'| = |\mathcal {T}|+|P| \le |\mathcal {T}|+2. \end{aligned}$$

The fact that $|\mathcal {T}| \ge 2|X|-3$ holds now follows by induction.

The fact that the bound is tight follows since for every $T \in B(X)$ there exists some triplet cover of *T* with cardinality $2|X|-3$ [e.g. a pointed cover Dress et al. (2012)]. $\square$

## A characterization of minimum triplet covers

In this section, we prove our main result, namely a characterization of minimum triplet covers in terms of the structure of their cover graphs. First, we recall that a graph $H=(V,E)$ is called a *2-tree* if there exists an ordering $v_1,v_2,\dots ,v_m$ of *V* such that $\{v_1,v_2\} \in E$ and, for $i=3,\dots ,m$, the vertex $v_i$ has degree 2 and belongs to a unique triangle in the subgraph induced by *H* on the set $\{v_1,v_2,\dots ,v_i\}$ (Guénoche et al. 2004, p. 235). It is easily seen that a 2-tree has treewidth at most 2, and conversely, every graph of treewidth at most 2 is a subgraph of a 2-tree.

### Theorem 1

Suppose that $\mathcal {T}$ is a triplet cover for a tree $T \in B(X)$. Then $\mathcal {T}$ is minimum triplet cover if and only if $\Gamma (\mathcal {T})$ is a 2-tree.

### Proof

Put $T=(V,E)$. Suppose that $\Gamma (\mathcal {T})$ is a 2-tree. Then since 2-trees on *n* vertices have $2n-3$ edges (Leclerc and Makarenkov 1998, p. 227) and $|X|=n$, we have $\mathcal {T}= 2|X|-3$. So $\mathcal {T}$ is a minimum triplet cover for *T*.

Conversely, suppose that $\mathcal {T}$ is a minimum triplet cover for some tree $T \in B(X)$. We shall prove that $\Gamma (\mathcal {T})$ is a 2-tree by induction on $n=|X|$. If $|X|=3,4$ it is clearly true. Suppose the statement holds for all *X* with $3 \le |X| \le n-1$.

Let $\mathcal {T}$ be a minimum triplet cover for *T* on *X* with $n=|X|$. Note that, by (M2), $\mu (\mathcal {T})$ equals 2 or 3. Also, note that $\mathcal {T}$ must be a minimal triplet cover for *T*.

**Fig. 3:**
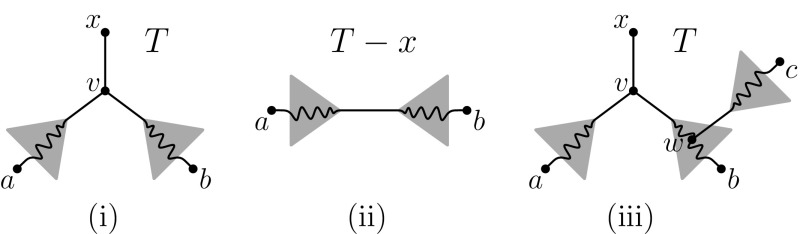
Figures for the proof of Theorem 1. **i** Leaf *x* and the other two leaves that form the triple in $S_v(\mathcal {T})$; **ii** the tree $T-x$ obtained from *T* by restricting this tree to $X-\{x\}$; **iii** the labelling of additional vertices in the case where $\mu (\mathcal {T})=3$. Squiggly lines denote paths in *T*

Suppose that $\mu (\mathcal {T})=2$. Let $x \in X$ be such that $\mu (x)=2$. Then there exist $a,b \in X-\{x\}$ with $xa, xb \in \mathcal {T}$. Consider the vertex $v\in V(T)$ adjacent to *x* in *T* (as shown in Fig. 3i). Then as $\mathcal {T}$ is a triplet cover, and *xa*, *xb* are the only elements in $\mathcal {T}$ containing *x*, it follows that $S_v(\mathcal {T}) = \{xab\}$.

Hence, $ab \in \mathcal {T}$. It follows that $\mathcal {T}':= \mathcal {T}-\{xa, xb\}$ is a triplet cover for $T-x$ (see Fig. 3ii) and since $|\mathcal {T}|= 2|X|-3$, it follows that $|\mathcal {T}'| = 2(|X|-1)-3$ and so $\mathcal {T}'$ is a minimum triplet cover for $T-x$. Since $T-x$ has one fewer leaf than *T*, we can apply the induction hypothesis and conclude that $\Gamma (\mathcal {T}')$ is a 2-tree. Then, since $\Gamma (\mathcal {T})$ is obtained from $\Gamma (\mathcal {T}')$ by attaching *x* to the endpoints of the edge $\{a,b\}$ in $\Gamma (\mathcal {T}')$, it follows that $\Gamma (\mathcal {T})$ is also 2-tree.

Now suppose that $\mu (\mathcal {T})=3$. We shall show that this is not possible, from which the theorem follows. Let $x \in X$ be such that $\mu (x)=3$ and let $v \in {V}$ denote the vertex adjacent to *x* in *T*. Then since $\mathcal {T}$ is a minimal triplet cover for *T* there must exist $a,b \in X-\{x\}$ distinct such that $xab\in S_v(\mathcal {T})$. Moreover, as $\mu (x)=3$ there must exist some $c \in X-\{x,a,b\}$ with $xc \in \mathcal {T}$. Since we also have $xa,xb\in \mathcal {T}$, and since $\mathcal {T}$ is a minimum triplet cover, it follows that $bc\in \mathcal {T}$.

Without loss of generality, assume *T* restricted to *x*, *a*, *b*, *c* is the quartet *xa*|*bc* (notice that we have symmetry involving *a* and *b*, and the quartet cannot be *xc*|*ab* because of the assumption that $xab \in S_v(\mathcal {T})$ where *v* is the vertex adjacent to *x* in *T*), as shown in Fig. 3iii. Let $w \in {V}$ be such that $w = \mathrm{med}(x,b,c)$.

We claim that $ac \not \in \mathcal {T}$. Assume for contradiction that $ac\in \mathcal {T}$. Since $\mathcal {T}$ is minimal and $xc\in \mathcal {T}$, there exists some vertex $u\in {V}$ and some $A\in S_u(\mathcal {T})$ such that $xc \subset A$. Note that as $\mu (x)=3$, we must have $u \in \{v,w\}$. If $u=v$ then $\mathcal {T}-\{xc\}$ is a smaller minimum triplet cover for *T* (since *v* is still supported by *abx*), and this contradicts the minimality of $\mathcal {T}$. Thus we may assume that $u=w$, in which case there is a set $A \in S_w(\mathcal {T})$ with $xc \subset A$. Since $\mu (x)=3$ and we already have $ax, bx, cx \in \mathcal {T}$ it follows that $A=xbc \in S_w(\mathcal {T})$ which implies that $bc \in \mathcal {T}$. However, as we already have $ab \in \mathcal {T}$, the additional assumption that $ac \in \mathcal {T}$ means that $\mathcal {T}-\{xc\}$ contains *ab*, *ac*, *bc* which provides an alternative set, namely *abc* in $S_w(T)$, in which case $\mathcal {T}-\{xc\}$ remains a triplet cover for *T*. But again this contradicts the minimality of $\mathcal {T}$. Thus, $ac \not \in \mathcal {T}$, as claimed.

Therefore, in summary, $xa,xb,xc,ab,bc \in \mathcal {T}$ and $ac \not \in \mathcal {T}$. We claim next that $\mathcal {T}' = \mathcal {T}- \{xb\} \cup \{ac\}$ is a triplet cover for *T*. Indeed, if *xb* is contained in some element in $S_u(\mathcal {T})$ for some $u \in {V}$, then since $\mu (x)=3$ we must have $u \in \{v,w\}$. Since $acx \in S_v(\mathcal {T}')$ and $abc \in S_w(\mathcal {T}')$ it follows that $\mathcal {T}'$ must be a triplet cover for *T*, as claimed.

To complete the proof, note that since $\mu _{\mathcal {T}'}(x)=2$, Lemma 2 implies $\deg _{\mathcal {T}'}(x)=1$. Hence, by (P5), $\mathcal {T}'^{-x}=\mathcal {T}' - \{xa,xc\}$ is a triplet cover of $T-x$. Since $T-x$ has one fewer leaf than *T* we can apply the induction hypothesis and conclude that the graph $\Gamma (\mathcal {T}'^{-x}) = (X-\{x\},\mathcal {T}'^{-x})$ is a 2-tree. Since any 2-tree has at least two vertices with degree 2 (Leclerc and Makarenkov 1998, p. 227), it follows that in $\Gamma (\mathcal {T}'^{-x})$ at least one of the two vertices *a* or *c* has degree 2 (since there cannot be a vertex $y \in X-\{x,a,b,c\}$ such that the degree of *y* in $\Gamma (\mathcal {T}'^{-x})$ is equal to 2 as, by assumption, $\mu (\mathcal {T})=3$). But if, without loss of generality, the degree of *a* in $\Gamma (\mathcal {T}'^{-x})$ is equal to 2, then $\mu _{\mathcal {T}}(a)=2$ must hold too which contradicts $\mu (\mathcal {T})=3$. This completes the proof. $\square$

The next result follows immediately from the last theorem and the fact that any 2-tree has at least two vertices with degree 2 [see e.g. Leclerc and Makarenkov (1998), p. 227]. It improves on the bound given in Proposition 2 (M2).

### Corollary 2

If $\mathcal {T}$ is a minimum triplet cover for some tree $T \in B(X)$ then $\mu (\mathcal {T})=2$.

Note that a 2-tree is a 2d-tree, but not necessarily conversely [Guénoche et al. (2004), Proposition 3.4] (a graph $G = (V, E)$ is called a *2d-tree* if there exists an ordering $x_1, x_2, \ldots , x_n$ of *V* such that $\{x_1, x_2\} \in E$ and, for $i=2, \ldots , n$ the vertex $x_i$ has degree 2 in the subgraph of *G* induced by $\{x_1, x_2, \ldots , x_i\}$). So Theorem 1 can be used to strengthen Theorem 1 of Huber and Steel (2014).

## Shellings

Given a triplet cover $\mathcal {T}$ of a tree $T \in B(X)$, we say that $\mathcal {T}$ is *T*-*shellable* if there exists an ordering of the elements in ${X \atopwithdelims ()2} - \mathcal {T}$, say $a_1b_1, a_2b_2, \dots , a_mb_m$ such that for every $1 \le i \le m$, there exists a pair $x_i,y_i$ of distinct elements in $X - \{a_i,b_i\}$ such that the restriction of *T* to the set $Y_i = \{a_i,b_i,x_i,y_i\}$ is the quartet $x_ia_i | y_ib_i$, and all elements in $Y_i \atopwithdelims ()2$ except $a_ib_i$ are contained in $\mathcal {T}_i = \mathcal {T}\cup \{a_jb_j \,: 1 \le j \le i-1\}$. If *T* is clear from the context then we sometimes just say that $\mathcal {T}$ is *shellable*, and we refer to the ordering of ${X \atopwithdelims ()2} - \mathcal {T}$ as a *shellable ordering*.

Although this combinatorial definition of shellability seems somewhat involved, its motivation rests on it being a sufficient condition for recursively determining the distances between all pairs of leaves (when the edges of *T* are assigned arbitrary positive edge lengths) starting with just the distance values for the pairs in the triplet cover. In other words, if a triplet cover $\mathcal {T}$ of a tree $T \in B(X)$ is shellable then the pairs of elements from *X* that are not already present in $\mathcal {T}$ can be ordered in a sequence so that the distance in *T* between the leaves in each pair is uniquely determined from the distances values on pairs that are either (i) present as an element of $\mathcal {T}$ or (ii) appear earlier in the sequence.

For example, for the tree *T* shown in Fig. 1i, and the triplet cover $\mathcal {T}$ consisting of the 7 pairs of elements of *X* that form the edges of $\Gamma (\mathcal {T})$ in Fig. 1iii, there are just three pairs from $$\left( {\begin{array}{c}X\\ 2\end{array}}\right) $$ that are not present in $\mathcal {T}$, namely *ad*, *ae*, *bd*. Ordering the pairs as $a_1b_1 = ae, a_2b_2=ad, a_3b_3=bd$ provides a shellable ordering, since for *ae* we can select $x_1y_1 = bc \in \mathcal {T}$ and observe that $x_1a_1|y_1b_1 = ba|ce$ is the quartet obtained by restricting *T* to $\{a,b,c,e\}$, the distance between $a_1=a$ and $b_1=e$ in *T* is determined uniquely by the five other distances involving pairs from $\{a,b,c,e\}$, and these five pairs are present in $\mathcal {T}$. Having determined the distance for $a_1b_1$ one can now use this (and the distances for pairs in $\mathcal {T}$) to compute the distance value for the pair $a_2b_2$ and, subsequently, for the pair $a_3b_3$.

We now gather together some facts concerning the shellability of triplet covers, including shellability of minimum triplet covers.

### Proposition 4

1. Suppose that $T\in B(X)$, $x\in X$, and $\mathcal {T}$ is a triplet cover of *T* such that $\mathcal {T}^{-x}$ is a triplet cover of $T-x$. If $\mathcal {T}^{-x}$ is ($T-x$)-shellable, then $\mathcal {T}$ is *T*-shellable.
2. Suppose that $\mathcal {T}$, $\mathcal {T}'$ are triplet covers of some tree $T \in B(X)$ and that $\mathcal {T}' \subseteq \mathcal {T}$. If $\mathcal {T}'$ is *T*-shellable, then so is $\mathcal {T}$.
3. If $\mathcal {T}$ is a minimum triplet cover for a tree $T \in B(X)$, then $\mathcal {T}$ is *T*-shellable.

### Proof

*(S1):* Put $T=(V,E)$. Suppose $x \in X$ such that $\mathcal {T}^{-x}$ is a triplet cover of $T-x$ which is shellable. Suppose that $v \in {V}$ is the vertex in *T* that is adjacent to *x* in *T*. Then there must exist $a,b \in X-\{x\}$ distinct with $xab\in S_v(\mathcal {T})$. Let $\mathcal {T}(x) = \{ de \in \mathcal {T}\,: \, x \in \{d,e\} \}$ and $\mathcal {T}^*(x) = \{ de \in {X \atopwithdelims ()2} \,: \, x \in \{d,e\} \text{ and } de \not \in \mathcal {T}(x) \}$, so that $\mathcal {T}= \mathcal {T}^{-x} \amalg \mathcal {T}(x)$ and

$$\begin{aligned} {X \atopwithdelims ()2} - \mathcal {T}= \left( {X-\{x\} \atopwithdelims ()2} - \mathcal {T}^{-x} \right) \amalg \mathcal {T}^*(x). \end{aligned}$$

Since $\mathcal {T}^{-x}$ is $(T-x$)-shellable, there is a shellable ordering of ${X-\{x\} \atopwithdelims ()2} - \mathcal {T}^{-x}$ so that all of the elements in that set can be added into $\mathcal {T}^{-x}$ to obtain ${X-\{x\} \atopwithdelims ()2}$.

To complete the shellable ordering it remains to add the elements of $$\left( {\begin{array}{c}X\\ 2\end{array}}\right) $$ that contain *x* to the ordering so far constructed. We consider two cases. First, suppose that neither $\{x,a\}$ nor $\{x,b\}$ form a cherry of *T*. Then for all $px \in \mathcal {T}^*(x)$, without loss of generality, the quartet induced by *T* on $\{x,a,b,p\}$ is *ap*|*xb*. Since we have that *xa*, *xb*, *ab* as $xab\in S_v(\mathcal {T})$ and also *ap* and *bp* as we have all elements in ${X-\{x\} \atopwithdelims ()2}$, it follows that we can add in *xp* as a next element of the shellable ordering. We can repeat this adding-in process for all remaining elements in $\mathcal {T}^*(x)$ (in any order) to obtain ${X \atopwithdelims ()2}$. So $\mathcal {T}$ is *T*-shellable in this case.

Second, suppose without loss of generality that $\{x,a\}$ forms a cherry. Then if $px \in \mathcal {T}^*(x)$, then the quartet induced by *T* on the set $\{x,a,b,p\}$ is *xa*|*bp*. So, using similar arguments as in the previous case, we can add in *xp* as a next element in the shellable ordering. It follows that we can repeat this process for all remaining elements in $\mathcal {T}^*(x)$ (in any order) to obtain a shellable ordering of ${X \atopwithdelims ()2}$. So $\mathcal {T}$ is *T*-shellable in this case too.

*(S2):* This follows immediately from the definition of shellability.

*(S3):* We proceed using induction on $n = |X|$. For $n=4$ the statement is clearly true. Suppose the statement is true up to and including $n-1 \ge 4$.

Let $\mathcal {T}$ be a triplet cover for some binary phylogenetic *X*-tree with $|X|= n$. By Corollary 2, $\mu (\mathcal {T}) =2$. Suppose that $x \in X$ with $\mu (x)=2$. Then, by Lemma 2, $\deg _{\mathcal {T}}(x)=1$. By (P4) it follows that $\mathcal {T}^{-x}$ is a triplet cover for $T-x$. Note that $\mathcal {T}^{-x}$ is minimum since $|\mathcal {T}^{-x}|= |\mathcal {T}|-2$. Thus by induction $\mathcal {T}^{-x}$ is $(T-x$)-shellable. Therefore, $\mathcal {T}$ is *T*-shellable by (S1). $\square$

### Corollary 3

For any tree $T \in B(X)$, suppose that $\mathcal {T}$ is a minimum triplet cover for *T*. Consider any assignment of strictly positive lengths to the edges of *T*, and the resulting assignment of inter-leaf distances on the pairs from $\mathcal {T}$. This function from $\mathcal {T}$ to ${\mathbb R}^{>0}$ uniquely determines *T* and its edge lengths, since no different tree $T' \in B(X)$ can induce the same inter-leaf distances on pairs from $\mathcal {T}$ under any positive weighting of the edges of $T'$.

### Proof

This follows immediately from Part (S3) of Proposition 4, combined with Theorem 6 of Dress et al. (2012). $\square$

Note that there are examples of sets $$\mathcal {T}\subseteq \left( {\begin{array}{c}X\\ 2\end{array}}\right) $$ having cardinality $2|X|-3$ that determine *T* and any set of positive edge lengths from inter-leaf distances, but which are not *T*-shellable (see Example 1).

### Example 1

**Fig. 4:**
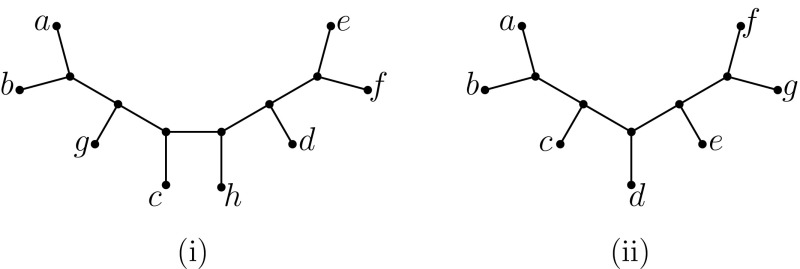
**i** A phylogenetic *X*-tree with $X=\{a,\ldots , h\}$. The set $\mathcal {T}=\{ab, ac, bc, cd, bd, ce, de, df, ef, ah, ag, fg, fh, gh\}$ is a minimal triplet cover but not a minimum one. The set $S_v(\mathcal {T})$ associated with each interior vertex *v* of *T* generates the following sequence (from left-most to right-most interior vertex): *abc*, *fgh*, *cbd*, *fgh*, *dec*, *edf*. (ii) A phylogenetic *X*-tree with $X=\{a\ldots , g\}$ for which the set $\mathcal {T}=\{ab,ad,bc,be,cd,cf,de,dg,ef,fg,ag\}$ determines *T* along with an assignment of positive edge lengths from the induced inter-leaf distances, yet $\mathcal {T}$ is not shellable

Put $X=\{a,b,c,d,e,f,g\}$ and let *T* be the caterpillar tree with exactly two cherries $\{a,b\}, \{f,g\}$ and intermediate leaves *c*, *d*, *e* (as shown in Fig. 4ii). Put $\mathcal {T}=\{ab,ad,bc,be,cd,cf,de,dg,ef,fg,ag\}$. Then $\mathcal {T}$ determines *T* and any set of positive edge lengths from inter-leaf distances, but it is not *T*-shellable (Dress et al. 2012, Example 6.2).

## Conclusion and open problems

As mentioned earlier, there are examples of minimal triplet covers $\mathcal {T}$ that are not minimum. The following provides a specific example.

### Example 2

Let $X=\{a,b,c,d,e,f,g,h\}$ and *T* be the phylogenetic *X*-tree having cherries $\{a,b\}, \{e,f\}$ and leaves, starting with cherry $\{a,b\}$, labeled in the order *g*, *c*, *h*, *d* (see Fig. 4i). Let

$$\begin{aligned} \mathcal {T}=\{ab, ac, bc, cd, bd, ce, de, df, ef, ah, ag, fg, fh, gh\}. \end{aligned}$$

Then $\mathcal {T}$ is a minimal triplet cover for *T*. Since $|\mathcal {T}|=14\not =2|X|-3$ it follows that $\mathcal {T}$ is not minimum.

An interesting problem would be to investigate the structure of the cover graph for minimal triplet covers.

Our results also suggest further questions for future work.

(i) There are formulae for counting the number of labeled 2-trees (Moon 1969). Is there a formula for counting the number of minimum triplet covers for a given phylogenetic *X*-tree?
(ii) We have shown that minimum triplet covers are shellable. It would be interesting to see how far this result extends. For example, is *every* triplet cover shellable? Understanding the structure of minimal triplet covers might help to shed light on this question.
